# Biochemistry and hematology parameters of the San Cristóbal Galápagos tortoise (*Chelonoidis chathamensis*)

**DOI:** 10.1093/conphys/coy004

**Published:** 2018-02-16

**Authors:** Gregory A Lewbart, John A Griffioen, Alison Savo, Juan Pablo Muñoz-Pérez, Carlos Ortega, Andrea Loyola, Sarah Roberts, George Schaaf, David Steinberg, Steven B Osegueda, Michael G Levy, Diego Páez-Rosas

**Affiliations:** 1 College of Veterinary Medicine, North Carolina State University, 1060 William Moore Drive, Raleigh, NC 27601, USA; 2 Galápagos Science Center, University San Francisco de Quito, Av. Alsacio Northia, Isla San Cristobal, Galápagos, Ecuador; 3 Dirección Parque Nacional Galápagos, Galapagos, Ecuador; 4 Department of Biology, University of North Carolina, Coker Hall, Chapel Hill, NC 27599, USA

**Keywords:** biochemistry, *Chelonoidis chathamensis*, Galapagos tortoise, health, hematology, lactate

## Abstract

As part of a planned introduction of captive Galapagos tortoises (*Chelonoidis chathamensis*) to the San Cristóbal highland farms, our veterinary team performed thorough physical examinations and health assessments of 32 tortoises. Blood samples were collected for packed cell volume (PCV), total solids (TS), white blood cell count (WBC) differential, estimated WBC and a biochemistry panel including lactate. In some cases not all of the values were obtainable but most of the tortoises have full complements of results. Despite a small number of minor abnormalities this was a healthy group of mixed age and sex tortoises that had been maintained with appropriate husbandry. This work establishes part of a scientific and technical database to provide qualitative and quantitative information when establishing sustainable development strategies aimed at the conservation of Galapagos tortoises.

## Teaser

The San Cristóbal Galapagos tortoise, *Chelonoidis chathamensis*, is native to San Cristóbal, the most eastern and oldest island of the Galapagos Archipelago. Although numerous studies on the ecology, behavior and genetics of Galapagos tortoises have been published, little is known about the health status of these animals. Here we report blood values from clinically healthy tortoises.

## Introduction

The San Cristóbal Galapagos tortoise, *Chelonoidis chathamensis*, is a species of chelonian native to San Cristóbal, the most eastern and oldest island of the Galapagos Archipelago, and the second settled by humans (Parent *et al.*, 2008). While the taxonomy of Galapagos tortoises has undergone a number of major shifts and revisions over the past several decades (Loveridge and Williams, 1957; Pritchard, 1996; Caccone *et al.*, 1999, 2002, 2004; Paquette *et al.*, 2012), it is currently widely accepted that 15 species are extant, and most of these are endemic to a specific island (Poulakakis *et al.*, 2015).

Although numerous studies on the ecology, behavior and genetics of Galapagos tortoises have been published, little is known about the health status of these animals. Peripheral blood chemistry and hematology parameters are useful diagnostic tools in health assessment and management in the reptiles (Geffre *et al.*, 2009; Zhang *et al.*, 2011; Gibbons *et al.*, 2013; Lewbart *et al.*, 2014; Muñoz-Pérez *et al.*, 2017) and many other taxa. To detect and monitor changes in the health of individuals and populations, it is paramount to determine species-specific normal values for blood parameters of interest. Reference intervals for many tortoise species exist (Marks and Citino, 1990; Gottdenker and Jacobson, 1995; Hart et al., 1991; López-Olvera *et al.*, 2003; Zaias *et al.*, 2006; Scope *et al.*, 2013; Eshar *et al.*, 2014; Andreani *et al.*, 2014; López *et al.*, 2017), in addition to recommended handling methods of blood films in the field (Sheldon *et al.*, 2016): characteristics that allow a comparative margin when generating new information.

In this study, we evaluated selected blood gas, biochemical, and hematology parameters along with vital signs and standard measurements from 32 semi-free-ranging Galapagos tortoises (*C. chathamensis*) at a captive breeding facility on the island of San Cristóbal. While this is an important component of a pre-introduction health assessment it should be noted that infectious disease screening might be warranted in some cases and can be a valuable part of such an effort.

## Materials and methods

### Ethics statement

This study was performed as part of a population health assessment authorized by the Galapagos National Park Service (GNP) through agreement and request from the GNP resident veterinarian Andrea Loyola and Diego Páez-Rosas; Juan Pablo Muñoz-Pérez from the Galápagos Science Center (GSC). Approved by the GSC ethics and animal handling protocol. All handling and sampling procedures were consistent with standard vertebrate protocols and veterinary practices.

### Study area

The Galapageura de Cerro Colorado is a captive giant tortoise breeding facility and sanctuary located on the northeast side of the San Cristóbal island, ~20 km from the town of Puerto Baquerizo Moreno. This ~12 hectares facility is home to ~140 free-ranging tortoises that are part of the breeding program or are progeny of the adults.

### Sampling

Captures and sampling were performed in March 2017 with the assistance and support of Galapagos National Park (PNG) personnel. Tortoises were located, positively identified by microchip, and selected for examination. This group was part of a program introducing tortoises to small farms (fincas) in the highlands of the San Cristóbal Island. This program, a collaborative effort between the PNG and local farmers, is designed to engage the farmers in conservation of a native species with the potential to damage crops. The hope is that tourism and the good will generated will result in mutual sustainability.

After a blood sample was obtained from each tortoise (see below), a thorough physical examination was performed including body temperature, morphometric measurements and fecal sample obtained (see below).

### Blood sample collection and handling

Blood samples were collected within an average of 5 min of capture. Blood samples of ~2.5 ml were obtained from either the brachial sinus or dorsal coccygeal vein using a heparinized (Heparin Sodium USP, 1000 units/ml; APP Pharmaceuticals, LLC, Schaumburg, IL, USA) 1.0 or 1.5 inch 22-gauge needle attached to a 3.0 ml syringe. The blood was then immediately divided into sub-samples which were used to make blood films on clean glass microscope slides, stored on ice in Eppendorf vials for laboratory analyses of manual hematocrit and total solids, and loaded into iSTAT cartridges or onto lactate strips within 10 min of sample collection.

### Blood gas and biochemistry parameters

An iSTAT portable clinical analyzer (Abbot Point of Care Inc., Princeton, NJ, USA) was used to obtain biochemistry, blood gas, and electrolyte results utilizing Chem8 cartridges (Abbot Point of Care Inc., Princeton, NJ, USA). The following parameters were measured and recorded: sodium (Na), potassium (K), chloride (Cl), ionized calcium (iCa), Total CO_2_, glucose (GLU), blood urea nitrogen (BUN), hematocrit (HCT), hemoglobin (Hb), anion gap and lactate. Blood lactate was determined using a portable Lactate Plus^TM^ analyzer (Nova Biomedical, Waltham, MA, USA).

### Hematology

Manual hematocrit (PCV%) was determined using high-speed centrifugation of blood-filled micro-hematocrit tubes and total solids (TS) via plasma analysis with a refractometer. Differential white blood differential counts were conducted by examining 100 white blood cells on a peripheral smear stained with Wright-Giemsa stain. The estimated total white blood cell counts were determined by averaging the number of white cells in 10 high-powered fields and multiplying by 2000 (Durbin *et al.*, 2009).

### Morphometric measures, body temperature and fecal sample collection

For each tortoise captured morphometric measurements were taken using a flexible 100 cm measuring tape. These included curved carapace length (CCL), curved carapace width (CCW), plastron length and shell depth. In addition, each tortoise was weighed with a precision of ±0.5 kg. Sex was determined in mature animals by plastron concavity and tail length. An EBRO^®^ Compact J/K/T/E thermocouple thermometer was used to obtain all temperature readings (model EW-91219-40; Cole-Parmer, Vernon Hills, IL, USA 60061). Core body temperatures were recorded from the cloaca using the probe EBRO^®^ T PVC epoxy tip 24GA × 1 m in length, which was inserted ~10 cm into the vent.

Respiratory rates per minute were measured by visualization and the heart rate with a Doppler ultrasound probe (Parks Medical Electronics, Inc., Aloha, OR, USA) in the area of the carotid or femoral artery.

Twenty-five of the tortoises defecated during handling. Fecal samples from these tortoises were collected, placed in Eppendorf tubes, and brought back to the laboratory for direct microscopic examination. A small fecal sample was placed on a slide with a coverslip and examined with a compound microscope using a 10× and 40× objective.

### Statistical analysis

We used ANOVA to test for differences in all morphometric, blood gas and hematology parameters between males, females and juveniles. We then ran post-hoc pairwise Wilcoxon rank sum tests when appropriate. A standard alpha of *P* = 0.05 was used for all statistical tests. We compared manually and iSTAT-derived hematrocrit values with a Passing-Bablok regression. All analyses were performed in R 3.4.1.

## Results

### Tortoise demographics and health status

The measurements sorted by sex and maturity are summarized in Table 1. There were significant sex- and age-related differences in all morphological traits: weight [ANOVA: *F*(2,29) = 59.38, *P* < 0.0001], curved carapace length [ANOVA: *F*(2,29) = 74.52, *P* < 0.0001], curved carapace width [ANOVA: *F*(2,29) = 57.95, *P* < 0.0001], carapace height [ANOVA: *F*(2,29) = 17.69, *P* < 0.0001], and plastron length [ANOVA: *F*(2,29) = 44.77, *P* < 0.0001]. In fact, males, females and juveniles differed significantly from one another in each of these traits, with *P* < 0.01 for all pairwise comparisons.

**Table 1:** Morphometric parameters from 32 semi-wild Galápagos tortoises (*Chelonoidis chathamensis*) at a breeding facility on San Cristóbal, Galápagos, Ecuador

Morphometric parameter	*N*	Median	Mean	SE	Min–Max
Weight (kg)*					
Male	15	85	87.27	5.29	50–115
Female	9	35	38.44	4.11	22–60
Juvenile	8	7.5	10.50	3.03	3–29
Curved carapace length (cm)*					
Male	15	106	106.17	3.19	85–124
Female	9	73	73.89	3.99	58–97
Juvenile	8	34	39.25	4.92	30–72
Curved carapace width (cm)*					
Male	15	103	105.03	2.79	87–122
Female	9	78	76.33	3.94	62–91
Juvenile	8	34.5	42.12	6.67	29–87
Carapace height (cm)*					
Male	15	28	31.73	3.42	21–77
Female	9	19	17.67	1.43	12–24
Juvenile	8	6	7.50	1.18	5–15
Plastron length (cm)*					
Male	15	70	67.80	2.84	37–82
Female	9	51.5	52.44	2.44	45–67
Juvenile	8	23.5	27.62	3.31	21–48

Asterisks indicate all groups are significantly different from one another.

All of the tortoises were bright, alert and responsive. Thirty-one of the 32 tortoises were deemed healthy enough for release to the fincas with the recommendation that one tortoise with an ocular discharge be reexamined by a veterinarian prior to release. Blood samples were drawn from a total of 32 animals but complete results were not obtained for every individual due to sample or analysis errors.

### Blood biochemical and hematology analysis

Tables 2 and 3 report the biochemistry, blood gas and hematology results. There were no statistically significant differences between males, females and juveniles in most of the blood chemistry and hematology parameters, including K [ANOVA: *F*(2,28) = 0.089, *P* = 0.915], iCa [ANOVA: *F*(2,28) = 1.411, *P* = 0.261], total CO_2_ [ANOVA: *F*(2,29) = 0.774, *P* = 0.47], GLU [ANOVA: *F*(2,29) = 1.174, *P* = 0.323], HCT [ANOVA: *F*(2,27) = 2.28, *P* = 0.122], Hb [ANOVA: *F*(2,23) = 1.304, *P* = 0.291], anion gap [ANOVA: *F*(2,28) = 0.353, *P* = 0.706], lactate [ANOVA: *F*(2,28) = 0.349, *P* = 0.709], PCV [ANOVA: *F*(2,22) = 0.04, *P* = 0.96], white blood cells [ANOVA: *F*(2,26) = 0.901, *P* = 0.481], heterophils [ANOVA: *F*(2,26) = 1.348, *P* = 0.277], lymphocytes [ANOVA: *F*(2,26) = 1.112, *P* = 0.344], eosinophils [ANOVA: *F*(2,26) = 0.597, *P* = 0.558] and basophils [ANOVA: *F*(2,26) = 1.931, *P* = 0.165]. Similarly, males and females did not differ in any hematology parameters, so we lumped them into a single ‘adult’ category for these traits (Table 3). However, we did find significant differences between some of the age/sex groups in Na [ANOVA: *F*(2,28) = 5.976, *P* = 0.007], Cl [ANOVA: *F*(2,28) = 7.278, *P* = 0.003], BUN [ANOVA: *F*(2,28) = 3.543, *P* = 0.043], and total solids [ANOVA: *F*(2,22) = 4.34, *P* = 0.026]. Post-hoc analyses showed that juveniles had lower total solids than adults (*W* = 103, *P* = 0.004), males had lower Na levels than juveniles (*W* = 14.5, *P* = 0.004), males had lower Cl levels than females (*W* = 28, *P* = 0.041) or juveniles (*W* = 15, *P* = 0.004), and juveniles had lower BUN levels than males (*W* = 93.5, *P* = 0.031). Manually determined hematocrit values were not systematically and proportionally different from iSTAT hematocrit values, with manually determined values being higher.

**Table 2:** Blood chemistry and blood gas parameters from 31 semi-wild Galápagos tortoises (*Chelonoidis chathamensis*) at a breeding facility on San Cristóbal, Galápagos, Ecuador

Blood chemistry parameter	*N*	Median	Mean	SE	Min–Max
Na* (mmol/l)					
Male	15	130.0	129.80	0.86	123–137
Female	8	132.0	131.38	1.18	125–135
Juvenile	8	134.5	134.75	1.10	131–141
K (mmol/l)					
Male	15	4.40	4.44	0.16	2.8–5.3
Female	8	4.35	4.36	0.15	3.6–4.9
Juvenile	8	4.35	4.35	0.19	3.6–5.1
Cl* (mmol/l)					
Male	15	99.0	98.40	0.88	92–104
Female	8	102.5	102.00	1.31	97–106
Juvenile	8	103.5	104.12	1.34	99–110
iCa (mmol/l)					
Male	15	1.48	1.48	0.03	1.24–1.64
Female	8	1.46	1.39	0.06	1.03–1.57
Juvenile	8	1.48	1.48	0.03	1.34–1.58
Total CO_2_ (mmHg)					
Male	15	26.0	26.33	1.16	20–38
Female	9	24.0	24.11	1.71	15–31
Juvenile	8	25.5	25.00	0.98	21–29
Glucose (mmol/l)					
Male	15	47	48.40	2.90	34–68
Female	9	43	44.22	4.01	21–66
Juvenile	8	51	52.12	2.55	44–66
Blood urea nitrogen* (mg/dl)					
Male	15	14.0	12.20	1.86	2–26
Female	8	9.0	9.38	1.80	2–18
Juvenile	8	4.5	5.25	1.26	2–11
HCT (%)					
Male	15	19	19.33	0.78	14–25
Female	7	16	16.86	0.91	14–21
Juvenile	8	16	17.00	1.28	14–25
HB (g/dl)					
Male	14	6.65	6.71	0.25	5.1–8.5
Female	7	5.80	6.00	0.29	5.1–7.1
Juvenile	5	5.80	6.32	0.58	5.4–8.5
anGAP (mmol/l)					
Male	15	10.0	10.33	0.88	5–19
Female	8	10.5	9.62	0.86	3–12
Juvenile	8	11.5	10.88	0.77	6–13
Lactate (mmol/l)					
Male	14	2.30	2.51	0.38	0.8–6.1
Female	9	1.80	2.10	0.21	1.4–3.0
Juvenile	8	1.45	2.12	0.61	0.3–5.4

The asterisks indicate significant differences between males and juveniles.

**Table 3:** Hematology values from a population of semi-wild Galápagos tortoises (*Chelonoidis chathamensis*) at a breeding facility on San Cristóbal, Galápagos, Ecuador

Hematological parameter	*N*	Median	Mean	SE	Min–Max
PCV (%)					
Adult	19	19.0	19.42	1.38	5–27
Juvenile	6	18.5	19.17	1.87	15–27
Total solids* (mg/dl)					
Adult	19	5.2	5.25	0.33	1.2–7.6
Juvenile	6	3.3	3.52	0.30	2.8–4.9
WBC (×10^9^/l)					
Adult	19	3150	3617.86	428.90	1400–7800
Juvenile	8	3850	4375.00	684.20	2800–8400
Heterophils (%)					
Adult	21	45.0	46.19	1.66	35–64
Juvenile	8	42.5	41.88	3.19	25–54
Lymphocytes (%)					
Adult	21	30	30.90	1.34	23–43
Juvenile	8	35	34.75	3.17	22–50
Monocytes (%)					
Adult	21	19	17.57	1.14	8–28
Juvenile	8	14	15.38	3.18	3–32
Eosinophils (%)					
Adult	21	1.0	1.57	0.32	0–6
Juvenile	8	1.5	2.00	0.65	0–5
Basophils (%)					
Adult	21	3.0	3.90	0.50	0–9
Juvenile	8	5.5	6.00	1.12	2–12

The asterisks indicate significant differences between adults and juveniles.

### Fecal analysis

No helminth ova or other parasites were observed on direct microscopic examination of feces from 25 tortoises.

## Discussion

Although many studies have reported baseline biochemical, blood gas and hematology values for other chelonian species (Gottdenker and Jacobson, 1995; Andreani *et al.*, 2014; Eshar *et al.*, 2014, 2016) nothing comprehensive has yet been published on Galápagos tortoises. Knafo *et al.* (2011) and Rivera *et al.* (2011) conducted studies based on sterilization of Galapagos tortoises and included PCV and total solids values on females and males. In both studies the PCV% and TS values are higher than those of our study. One difference is the mean TS of the females was 7.8 g/dl in Knafo *et al.* (2011) and that of our females was 4.8 g/dl. Blake *et al.* (2015) measured PCV and TS values for 37 wild adult female tortoises (*Chelonoidis porteri*) on Santa Cruz in two locations (Cerro Fatal and La Reserva) as part of a feeding ecology study. While the both PCV and TS were positively correlated with elevation, the differences were not significant. The average mean PCV and TS for the two groups was 20.9% and 7.9 mg/dl, respectively. This PCV value is just slightly higher than the tortoises in our study but the TS is much higher. This could be related to the reproductive status of the females. Knafo *et al.* (2011) collected their samples in November, and Blake *et al.* (2015) collected their samples in November and December, which is at the end of the mating season, and the beginning of the nesting season. Our samples were obtained in the middle of the mating season but several months before nesting begins (MacFarland *et al.*, 1974a, 1974b; Rostal *et al.*, 1998). While a definitive explanation for this discrepancy is unknown, another possibility includes intrageneric differences between *C. chathamensis*, *C. porteri* and *C. nigra*. However, Rivera *et al.* (2011) report that males of *C. nigra* have similar TS values in relation to the males monitored in our study (5.6 and 5.2 g/dl, respectively), so the interspecific difference between males is much smaller than between females.

We judged the tortoises we examined to be clinically healthy and the hematology (total white cell/differential counts) and blood chemistry values were generally consistent with those reported for other healthy tortoise species (Marks and Citino, 1990; Gottdenker and Jacobson, 1995; López-Olvera *et al.*, 2003; Zaias *et al.*, 2006; Scope *et al.*, 2013; Eshar *et al.*, 2014; Andreani *et al.*, 2014; López *et al.*, 2017). One exception was the PCV%, which on average was lower in the Galapagos tortoises compared to desert tortoises, *Gopherus agassizii*, (Christopher *et al.*, 1999), Hermann’s tortoises, *Testudo hermanni* (Andreani *et al.*, 2014), ploughshare tortoises, *Astrochelys yniphora* (López *et al.*, 2017), radiated tortoises, *Geochelone radiata* (Zaias *et al.*, 2006), and African spurred tortoises, *Geochelone sulcata* (Eshar *et al.*, 2016). This could be related to species size, as the next lowest mean PCV% of this group was the sulcata, second largest of the included species. A finding relevant to future methodologies is that the iSTAT proved accurate in determining PCV in Galapagos tortoises. In some animals iSTAT hematocrit values are lower than the manually obtained PCV%. These species include loggerhead sea turtles (Wolf *et al.*, 2008), rainbow trout (Harter *et al.*, 2014), marine iguanas (Lewbart *et al.*, 2015) and Quaker parrots (Rettenmund *et al.*, 2014). Thus, for Galápagos tortoises, the Chem 8 cartridge used with an iSTAT reader provided an accurate estimation of the PCV%.

The mean total white blood cell counts (WBC) of the Galapagos tortoises in our study were lower than those reported in 42 wild Santa Cruz Galapagos tortoises (Sheldon *et al.*, 2016). This robust study examined several methods of determining the WBC, including an estimated count using multiple fields with a 40× objective (Sheldon *et al.*, 2016). While there was overlap with the ranges, their mean total count was 8.85 × 10^3^ cells/μl, compared to our mean of 3.79 × 10^3^ cells/μl. The San Cristóbal Galápagos tortoise WBC values were just slightly lower than those reported for other tortoise species (Christopher *et al.*, 1999; Zaias *et al.*, 2006; López *et al.*, 2017). With so many variables between the species, like age, size, and time of year, making direct comparisons with differential counts is challenging. One noteworthy finding was that the San Cristóbal Galapagos tortoises had higher numbers of monocytes than the Santa Cruz tortoises and other species in the family Testudinidae. This fact might be worth pursuing with a larger and more comprehensive hematology study.

The small sample size of our study precluded the calculation of formal reference intervals, a process that would require a minimum of 120 individuals (Campbell, 1995; Geffre *et al.*, 2009; Friedrichs *et al.*, 2012). In addition, all of the samples were obtained on one day; thus, the possibility exists that sampling at different times of the year would yield different results (Campbell, 2006; Zaias *et al.*, 2006; Scope *et al.*, 2013). This was the case with a population of radiated tortoises (*G. radiata*) on St. Catherine’s Island, GA, USA (Zaias *et al.*, 2006) that was sampled in both January and August of the same year.

Although direct fecal examination of 25 animals did not reveal any helminth parasites or ova, this does not represent a comprehensive parasite screening, as there are far more sensitive tests that were not undertaken for logistical and cost reasons.

In summary, data reported in this study represent an important step toward determining a baseline range of values against which future blood gas and biochemistry results in Galapagos tortoises can be compared. Future research should work toward establishing reference values in the other *Chelonoidis* species, as well as expanding sampling to facilitate comparisons of blood values across age groups and seasons.
